# A Review of Cyclophosphamide-Induced Transplantation Tolerance in Mice and Its Relationship With the HLA-Haploidentical Bone Marrow Transplantation/Post-Transplantation Cyclophosphamide Platform

**DOI:** 10.3389/fimmu.2021.744430

**Published:** 2021-09-29

**Authors:** Hisanori Mayumi

**Affiliations:** Mayumi GP & Cardiology Clinic, Saitama City, Japan

**Keywords:** cyclophosphamide-induced tolerance, drug-induced tolerance, haploBMT/PTCy, haploBMT, PTCY, PTCy-haplo HSCT, clonal destruction, clonal deletion

## Abstract

The bone marrow transplantation (BMT) between haplo-identical combinations (haploBMT) could cause unacceptable bone marrow graft rejection and graft-versus-host disease (GVHD). To cross such barriers, Johns Hopkins platform consisting of haploBMT followed by post-transplantation (PT) cyclophosphamide (Cy) has been used. Although the central mechanism of the Johns Hopkins regimen is Cy-induced tolerance with bone marrow cells (BMC) followed by Cy on days 3 and 4, the mechanisms of Cy-induced tolerance may not be well understood. Here, I review our studies in pursuing skin-tolerance from minor histocompatibility (H) antigen disparity to xenogeneic antigen disparity through fully allogeneic antigen disparity. To overcome fully allogeneic antigen barriers or xenogeneic barriers for skin grafting, pretreatment of the recipients with monoclonal antibodies (mAb) against T cells before cell injection was required. In the cells-followed-by-Cy system providing successful skin tolerance, five mechanisms were identified using the correlation between super-antigens and T-cell receptor (TCR) Vβ segments mainly in the H-2-identical murine combinations. Those consist of: 1) clonal destruction of antigen-stimulated-thus-proliferating mature T cells with Cy; 2) peripheral clonal deletion associated with immediate peripheral chimerism; 3) intrathymic clonal deletion associated with intrathymic chimerism; 4) delayed generation of suppressor T (Ts) cells; and 5) delayed generation of clonal anergy. These five mechanisms are insufficient to induce tolerance when the donor-recipient combinations are disparate in MHC antigens plus minor H antigens as is seen in haploBMT. Clonal destruction is incomplete when the antigenic disparity is too strong to establish intrathymic mixed chimerism. Although this incomplete clonal destruction leaves the less-proliferative, antigen-stimulated T cells behind, these cells may confer graft-versus-leukemia (GVL) effects after haploBMT/PTCy.

## Introduction

Historically the best results of human allogeneic BMT have been obtained when the donor is an HLA-matched sibling or an unrelated donor who is matched to the recipient at each of eight high-expression HLA molecules: both alleles at each of HLA-A, -B, -C and -DRB1. An HLA-haploidentical related donor is one who shares one HLA-haplotype, including one allele each of HLA-A, -B, -C, and -DRB1 with the recipient by common inheritance but is mismatched for a variable number of HLA alleles on the unshared HLA haplotype (1, 2). These mismatches on the unshared HLA haplotype are associated with unacceptable bone marrow graft rejection (host-versus-graft-reaction: HVGR) and acute and chronic graft-versus-host disease (GVHD) (1, 2).

**Figure 1:**
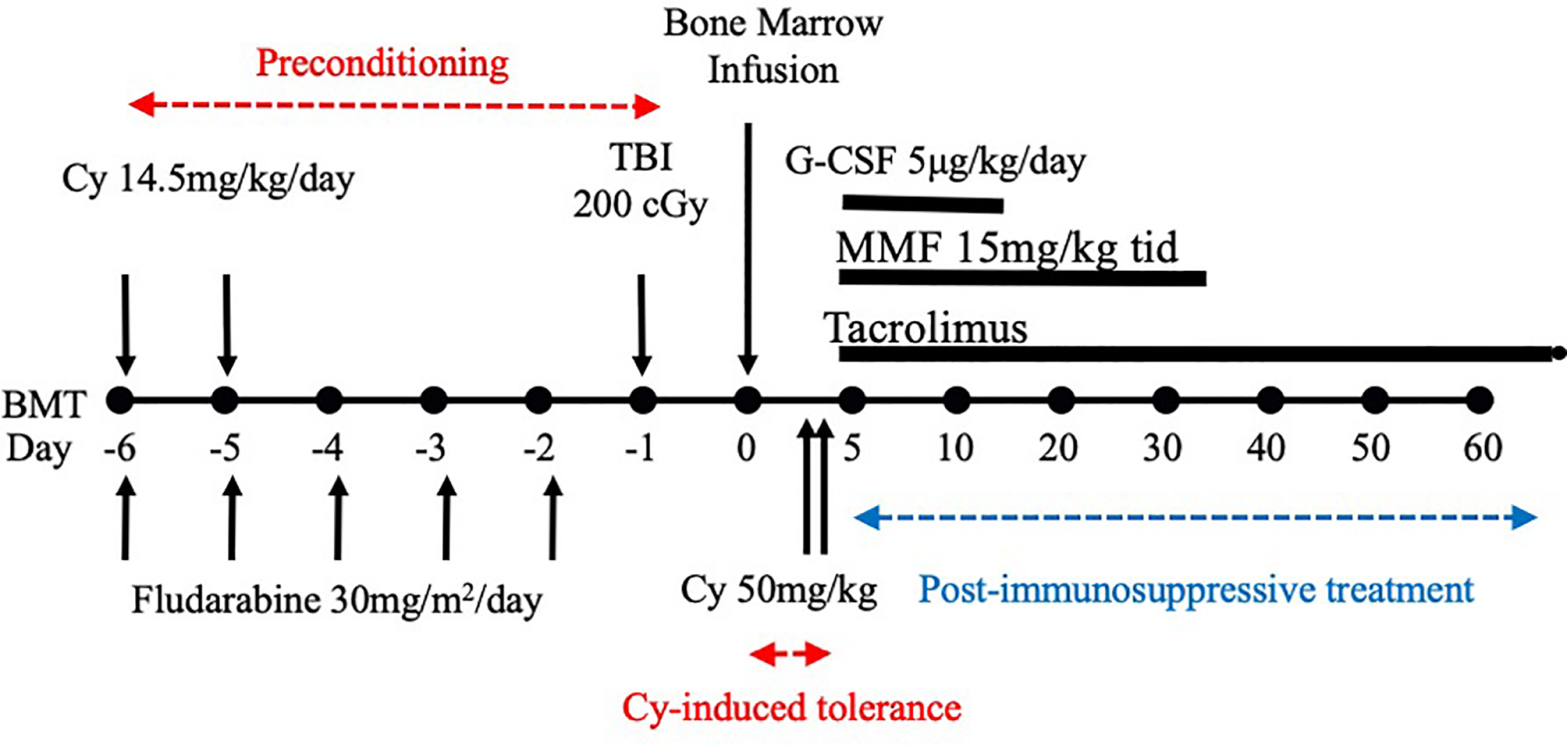
Treatment schema for nonmyeloablative, HLA-haploidentical bone marrow transplantation (haploBMT) with high-dose, post-transplantation cyclophosphamide (PTCy). The protocol comprises nonmyeloablative pretreatment with fludarabine 30 mg/m2/day on days -6 through -2, Cy 14.5 mg/kg/day on days -6 and -5, total body irradiation (TBI) with 200 cGy on day -1 (=all of these are preconditioning), and BMT on day 0 followed by Cy 50 mg/kg on days 3 and 4 (=Cy-induced tolerance). The immunosuppressive drugs that may suppress T or B cell proliferation (=MMF and tacrolimus) are required to be used after the Cy-induced tolerance protocol (=Post-immunosuppressive treatment). This methodology was effective in crossing the haplo-identical thus only 1-3 HLA plus minor histocompatibility antigen mismatched barriers of human BMT, and was spread all over the world as a typical platform of the HLA-haploidentical BMT/PTCy method. This platform represents the clinical fruition of our Cy-induced tolerance system. Cy, cyclophosphamide; BMT, bone marrow transplantation; MMF, mycophenolate mofetil; TBI, total body irradiation; G-CSF, granulocyte-colony stimulating factor.

After we published the novel Cy-induced tolerance method, using a single bolus dosing of Cy, that can overcome skin allograft barriers in H-2 identical murine combinations (3), this method was re-evaluated in a mouse BMT model by Johns Hopkins Hospital group (4), and soon used for human BMT to cross HLA-haploidentical combinations (1, 2). Their protocol in humans (**Figure 1**) comprises nonmyeloablative pretreatment with fludarabine 30 mg/m^2^/day on days -6 through -2, Cy 14.5 mg/kg/day on days -6 and -5, total body irradiation (TBI) with 200 cGy on day -1 (=preconditioning), and BMT on day 0 followed by Cy 50 mg/kg on days 3 and 4 (=Cy-induced tolerance), and tacrolimus and mycophenolate mofetil from day 5 (=Post-immunosuppressive treatment) (1, 2). This methodology was effective in crossing the haploidentical barrier in human BMT, and was spread all over the world as a typical platform of the HLA-haploidentical BMT (haploBMT)/PTCy method.

An activated form of cyclophosphamide, phosphoramide mustard, alkylates, or binds, to DNA and its cytotoxic effect is mainly due to cross-linking of strands of DNA and RNA, and to inhibition of protein synthesis as was shown by A.C. Aisenberg (5). Administration of high dose Cy early after haploBMT selectively kills proliferating, alloreactive T cells while sparing non-alloreactive T cells responsible for immune reconstitution and resistance to infection (2). Thus, hospitalization for haploBMT/PTCy is unnecessary for more than 80% of the cases at Johns Hopkins Out Patient Clinic (E.J. Fuchs; personal communication). Haploidentical BMT with high-dose PTCy is now becoming a safe, effective and inexpensive treatment for patients with hematologic malignancies or hemoglobinopathies and for the tolerance induction to transplants of solid organs (6) from the same donor (2). The successful application of the Cy-induced tolerance method to cross the HLA barrier in allogeneic hematopoietic cell transplantation brings to fruition decades of effort, starting with Morris Berenbaum (7) and continued in Fukuoka, Japan (8–10) to achieve transplantation tolerance in the clinic.

To further understand Cy-induced tolerance, it may be useful to provide the historical context of the Cy-induced tolerance system developed in mice more than 30 years ago. We had already shown, mainly in H-2 identical strain combinations, the sequential five mechanisms of skin allograft tolerance consisting of: 1) the clonal destruction of antigen-stimulated-thus-proliferating mature T cells with Cy; 2) the peripheral clonal deletion associated with immediate peripheral chimerism; 3) the intrathymic clonal deletion associated with intrathymic chimerism; 4) the delayed generation of Ts cells; and 5) the delayed generation of clonal anergy (8–10). Here, the “clonal deletion” means the clonal cell death due to the apoptosis caused by the cell contact alone with responsible antigens. Recent studies, however, emphasize the importance of the immediate generation of Ts cells (i.e. regulatory T cells) rather than clonal destruction of the effector T cells (11–14). In the present review, therefore, I will reevaluate the transfer experiments that we had repeatedly performed to detect host Ts cell or factor activities against donor antigens.

Another reason for the great success of haploBMT/PTCy may be ascribed to the GVL effect due to the split tolerance generated after Cy-induced tolerance (8–10). In the fully allogeneic murine donor→recipient combination of C57BL/6 (B6; H-2^b^)→AKR/J (AKR; H-2^k^), tolerance to the EL-4 tumor (originated from B6) was induced only when 40 x 10^6^ live B6 spleen cells (SC) were injected *i.v.* into recipient AKR mice on day 0 followed by a single dose of 150mg/kg Cy *i.p.* on day 1, 2, or 3. However, the recipient AKR mice that tolerated the EL-4 tumor were nevertheless able to reject skin allografts from B6. This phenomenon was attributed to the persistence of a subset of alloreactive T cells, which were potentially less proliferative than the T cells that underwent clonal destruction, and appears when the antigenic disparity between the donor and recipient is strong (8–10). Since the histocompatibility (H) antigen mismatches in the HLA-haploidentical BMT/PTCy appear to be 1-3 HLA plus minor H antigens, a certain amount of this split tolerance mechanism works. A part of mature T cells in the recipient appears to be less proliferative against the antigen stimulation, mature quickly within 1-3 days before Cy-treatment, and thus remains in an anamnestic state after the Cy-treatment. In the haploBMT/PTCy patients, therefore, the small amount of GVL effect is naturally prepared, depending on the H antigen disparity and BMT-Cy-injection interval.

At the end of the review, moreover, I will introduce an application of this system to xenogeneic combination.

## Preliminary Study in Cy-Induced Tumor-Allograft Tolerance and Failure in Skin-Allograft Tolerance to Cross Fully Allogeneic Murine Combinations

Drug-induced tolerance, comprising a combination of an antigen and an antimitotic drug, was intensively studied in many laboratories in the 1960s and 1970s (5, 7, 15–23). Clinically applicable methods to achieve a long-lasting solid organ tolerance, however, had not been established by a short course of the antigen plus immunosuppressive drug treatment (5, 7, 15–23).

The preliminary study in Cy-induced tolerance was started at Prof. K. Nomoto’s laboratory (Fukuoka, Japan) by Dr. T. Shin (24) obtaining hints from the foregoing studies (5, 7, 15–23) and the guidance from Associate Prof. K. Himeno in 1982. In the fully allogeneic murine donor→recipient combination of C57BL/6 (B6; H-2^b^)→AKR/J (AKR; H-2^k^), tolerance to the EL-4 tumor (originated from B6) was induced only when 40 x 10^6^ live B6 spleen cells (SC) were injected *i.v.* into recipient AKR mice on day 0 followed by a single dose of 150mg/kg Cy *i.p.* on day 1, 2, or 3. Neither SC alone nor Cy alone could induce tolerance. Tolerance induced this way continued for more than 10-14 weeks, and was antigen-specific because the third-party tumor Meth-A fibrosarcoma (H-2^d^) from BALB/c mice (BALB: H-2^d^) was rejected in normal fashion in the AKR mice (H-2^k^) made tolerant of B6 (H-2^b^) (24).

**Figure 2:**
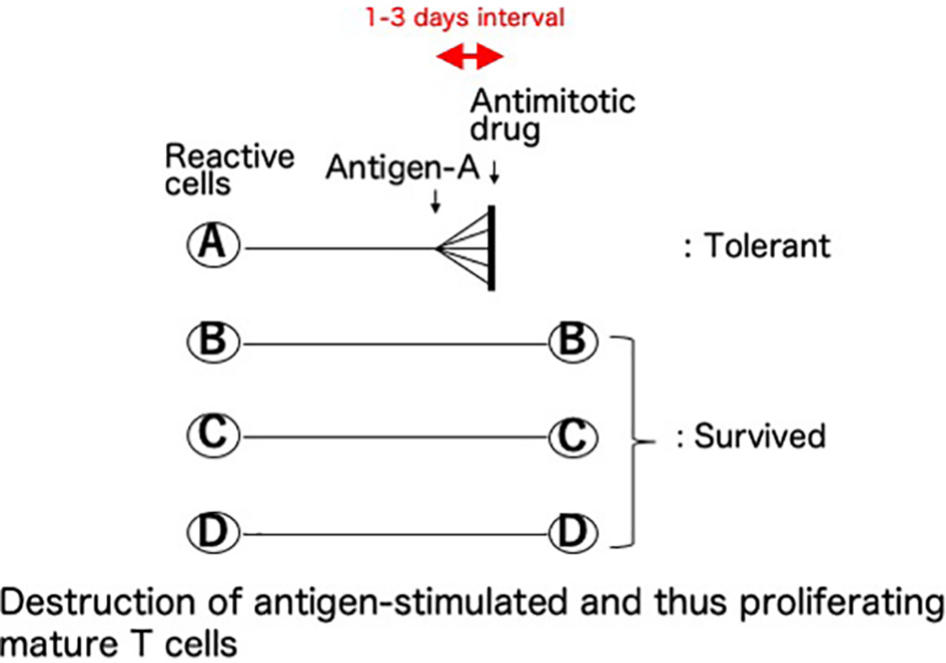
Clonal destruction as the central mechanism of cyclophosphamide-induced tolerance. This basic mechanism of the cells-followed-by-Cy system was named as “clonal destruction”. This most important mechanism is considered to be the destruction, with Cy, of the antigen-stimulated, and thus-proliferating, cells. Namely, the mature T (or B) cells reactive against the allo-antigens clonally expand after the injection of allogeneic cells. The DNA contained in the proliferating blast cells is especially sensitive to Cy, an alkylating agent, and thus the clones are selectively destroyed with this agent given 1-3 days later while leaving the other resting clones intact. The term “clonal destruction” is preferably used to segregate this mechanism from peripheral or intra-thymic clonal deletion. In Cy-induced tolerance *in vivo*, clonal destruction of antigen-reactive T cells occurs in both directions. Since the infused donor cells are alive, the mixed lymphocyte reactions occur in both directions between the donor lymphocytes and the recipient lymphocytes, and subsequently the reactive clones among both the host cells and the infused donor cells are destroyed by Cy while leaving the other resting clones intact.

This basic mechanism of the cells-followed-by-Cy system was named (8–10) as “clonal destruction” (**Figure 2**). This mechanism was considered to be the destruction, with Cy, of the antigen-stimulated, and thus-proliferating, mature T (or B) cells reactive against the allo-antigens expressed by the injected allogeneic cells (8–10). The DNA contained in the proliferating blast cells is especially sensitive to Cy and thus the clones are selectively destroyed with this agent while leaving the other resting clones intact (3). The clonal destruction mechanism is independent of the bone marrow and thymus and works only on mature T (or B) cells (8–10), as confirmed *in vitro* (25). In contrast to the cells-followed-by-Cy system, the Cy-followed-by-cells system (8–10) does not involve clonal destruction (8–10).

The allograft tolerance established in the fully allogeneic donor-recipient combination of B6 (H-2^b^)→AKR (H-2^k^) appeared to be promising as long as tumors were used as the allografts, but not when trunk skin grafts were sutured precisely on the graft beds preserving the panniculus carnosus (26). The B6 skin was rejected in a normal (or sometimes even an accelerated) fashion in the AKR recipients made tolerant of B6 (27).

## The First Cy-Induced Skin-Allograft Tolerance in H-2-Identical Murine Combinations

**Figure 3:**
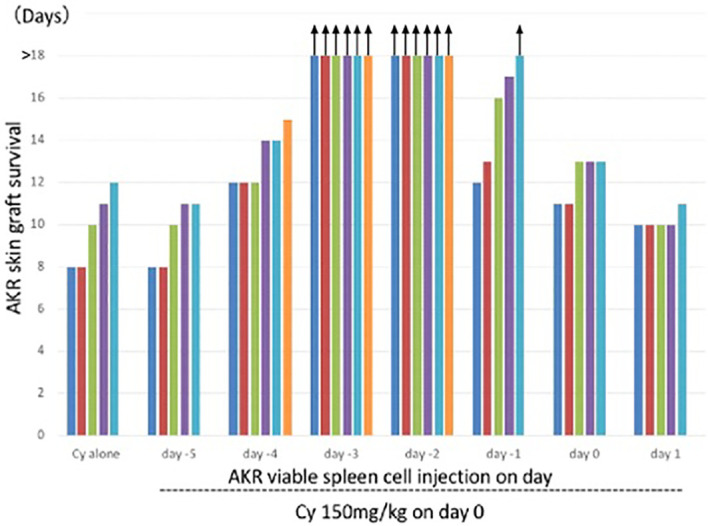
Optimal timing of Cy treatment for skin allograft tolerance induction in an H-2 identical murine combination. C3H mice (H-2^k^) were primed with viable 50 x 106 AKR (H-2^k^) spleen cells on day -5, -4, -3, -2, -1, 0, or 1 and treated with 150mg/kg Cy on day 0. A control group given Cy alone was set up. Grafting with AKR skin was carried out on day 7. Each bar represents graft survival (in days) in each mouse. All mice were killed for other assays on day 25. Therefore, the bars with arrows indicate which grafts were viable on day 25 and which were presumed thereafter. Original data derived from reference #3 (3).

A complete skin allograft acceptance associated with excellent hair growth was first obtained when the donor and recipient were H-2 identical and differed only in minor H antigens (3, 28). The optimal timing of 150mg/kg Cy treatment for tolerance induction in the C3H mice (H-2^k^), determined by AKR (H-2^k^) skin grafting, was 2 or 3 days after the *i.v.* injection of 50 x 10^6^ AKR (H-2^k^) live SC (3) (**Figure 3**). From our repeated experiments of Cy-induced tolerance, the optimal timing of cell injection and Cy-treatment was considered to be 1-3 days (8–10).

Relevant to allogeneic blood or marrow transplantation, the tolerance induction in the H-2 identical murine combination completely suppressed the graft-versus-host (GVH) reaction (29). When the SC from the AKR mice made tolerant of C3H were used to reconstitute the lethally irradiated C3H mice, the recipient C3H survived without any signs of GVHD (29). Interestingly, the AKR mice made tolerant of C3H were already minimally mixed chimeric as was shown in the chimeric analysis of the thymus cells in the AKR mice after tolerance induction with C3H SC plus Cy (29).

Another important suggestion from this result was that the clonal destruction induced in the recipient mice was bidirectional (29). Since the infused donor cells are alive, the mixed lymphocyte reactions occur in both directions between the donor lymphocytes and the recipient lymphocytes, and subsequently the reactive clones are destroyed in both the donor and recipient lymphocytes with Cy at the same time (29–31).

The optimal tolerogen was SC given intravenously (100 x 10^6^/mouse), which is the maximal dose collectable from a single donor mouse (3), followed by 200mg/kg Cy in the H-2 matched murine combinations. Viable cells are indispensable for a long-lasting tolerance (3, 8–10, 24, 28), because the two main factors required for the tolerogen are both antigenicity and hematopoietic capability (3, 8–10, 28). A solid organ graft itself may not be a suitable and sufficient tolerogen (8–10, 24), because antigen dissemination, and synchronous proliferation of the reactive T cells, throughout the entire body are required for efficient clonal destruction (**Figure 2**). For the same reason, a subcutaneous route for cell injection is not effective in inducing profound tolerance (8–10, 24).

As described above, the optimal timing of Cy-treatment is day 2 or 3 (cell injection = day 0), when a single dose of Cy is used (3). Treatment with cyclosporine A (31, 32) or steroids (33) before or together with the cell injection prevents the development of the tolerance induction through the clonal destruction, because the cell proliferation is blocked with these drugs used for pretreatment. If cyclosporine, steroids, tacrolimus, or other immunosuppressive drugs that may suppress T or B cell proliferation are required to be used in combination with the antigens-followed-by-antimitotic drug system, they should be given after the treatment with the antimitotic drugs including Cy (31, 32), as in the Johns Hopkins platform (**Figure 1**).

## Cyclophosphamide-Induced Tolerance to Allogeneic Major H Antigens

Although a completely tolerant state with luxurious hair growth was consistently maintained for more than 100 days in the recipient mice made tolerant of MHC-matched combinations with 100 x 10^6^ allogeneic SC injection followed 2-3 days later by 200 mg/kg Cy, such a tolerant state had never been induced in an MHC-mismatched fully allogeneic combination before 1986, when H. Mayumi started further studies in Cy-induced tolerance in the laboratory of Dr. Robert A. Good in St. Petersburg, Florida.

a. Less Proliferative Quick Maturation of the Reactive Clones and Resulting Split Tolerance Stage

**Figure 4:**
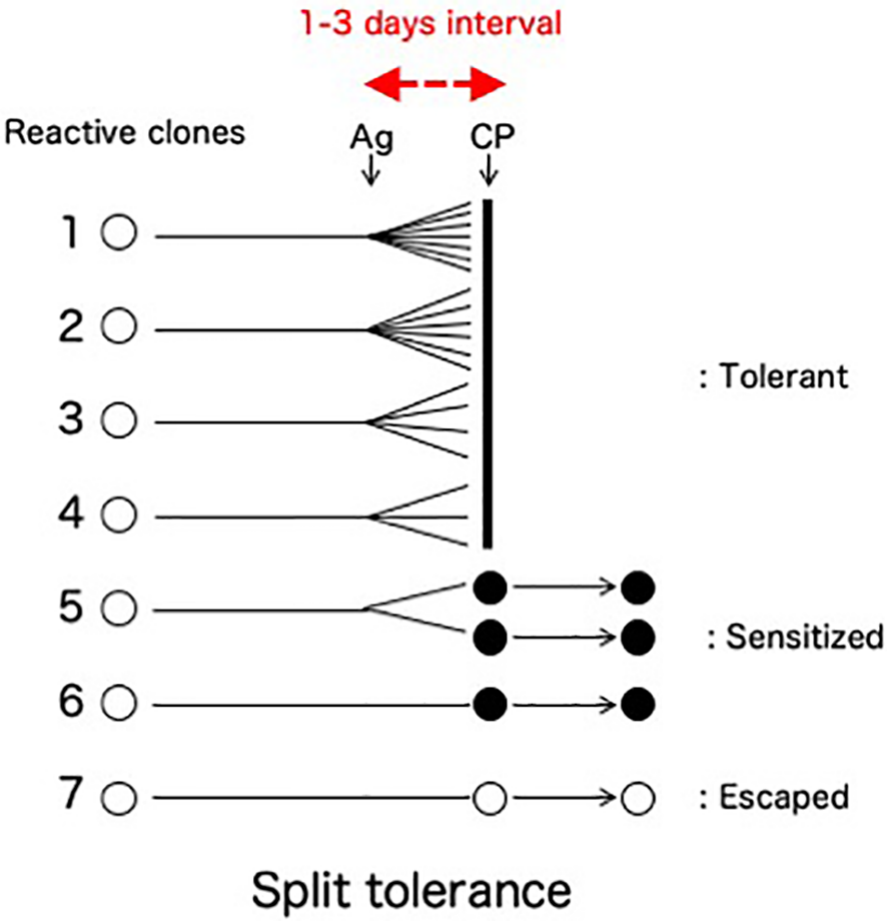
Split tolerance due to less proliferative quick maturation. A fraction of mature T cells in the recipient is less proliferative against the antigen stimulation, mature quickly before the Cy-treatment given 1-3 days later, and thus remain in an anamnestic state after the Cy-treatment. Interestingly, such anamnestic memory T cell activities are not augmented by the subsequent immunization with responsible allogeneic SC, skin allografting, or tumor allografting in the absence of suppressor T cells (34). The destroyed cell population (Clones 1-4) and the population left behind in an anamnestic state (Clones 5-6) may be different in their capacity for clonal expansion.

A clinically important feature of the cells-followed-by-Cy system is the fate of the mature T cells that are reactive against donor antigens but escape from clonal destruction with Cy (**Figure 4**). From our previous study *in vivo* (8–10, 34) and *in vitro* (25, 35), a fraction of mature T cells in the recipient (or the responder cells in the one-way mixed lymphocyte culture) proliferated less to the stimulating alloantigen, matured quickly before the Cy (or 5-fluorouracil *in vitro*)-treatment, and thus remained in an anamnestic state after the Cy (or 5-fluorouracil)-treatment. Here, the mixed lymphocyte reaction (MLR) was completely suppressed after the tolerance induction (24, 25, 35), but anamnestic reactions were manifested as a second set rejection of both skin allografts and tumor allografts of small doses (27, 36) or as a second set reaction in both delayed footpad reactions and cytotoxic T lymphocyte (CTL) activity (34). The T cells responsible for these anamnestic effects presumably escaped the destruction with Cy by maturating quickly within the 1-3 days interval between the SC and Cy-treatment (34). The greater the antigenic disparity between donor and host, the larger number of sensitized cells remained after tolerance induction by the cells-followed-by-drug system (8–10, 27). The existence of such anamnestic cells in the tolerant recipient (or cultured cells) is manifested as a form of split tolerance (8–10, 24, 25, 27, 34–36): that is, both second-set rejection of a skin allograft and complete acceptance of a tumor allograft occur in the same mouse *in vivo* (8–10, 24, 34, 36, 37). Furthermore, even if a large dose of allogeneic tumor cells is accepted in the tolerant mice, a small dose of the same tumor is rejected in a second-set fashion (27). *In vitro*, complete suppression of CTL activity and MLR was associated with anamnestic interleukin-2 production (25). An unexpected feature of the T cells left behind after incomplete clonal destruction is that such anamnestic memory T cell activities could not be augmented by immunization with responsible allogeneic SC, skin allografting, or tumor allografting (34).

b. Tolerance Induction Across Various Allogeneic Barriers; Genetic Analysis of Sensitivity of Tolerance Induction Using Congenic Strains

**Figure 5:**
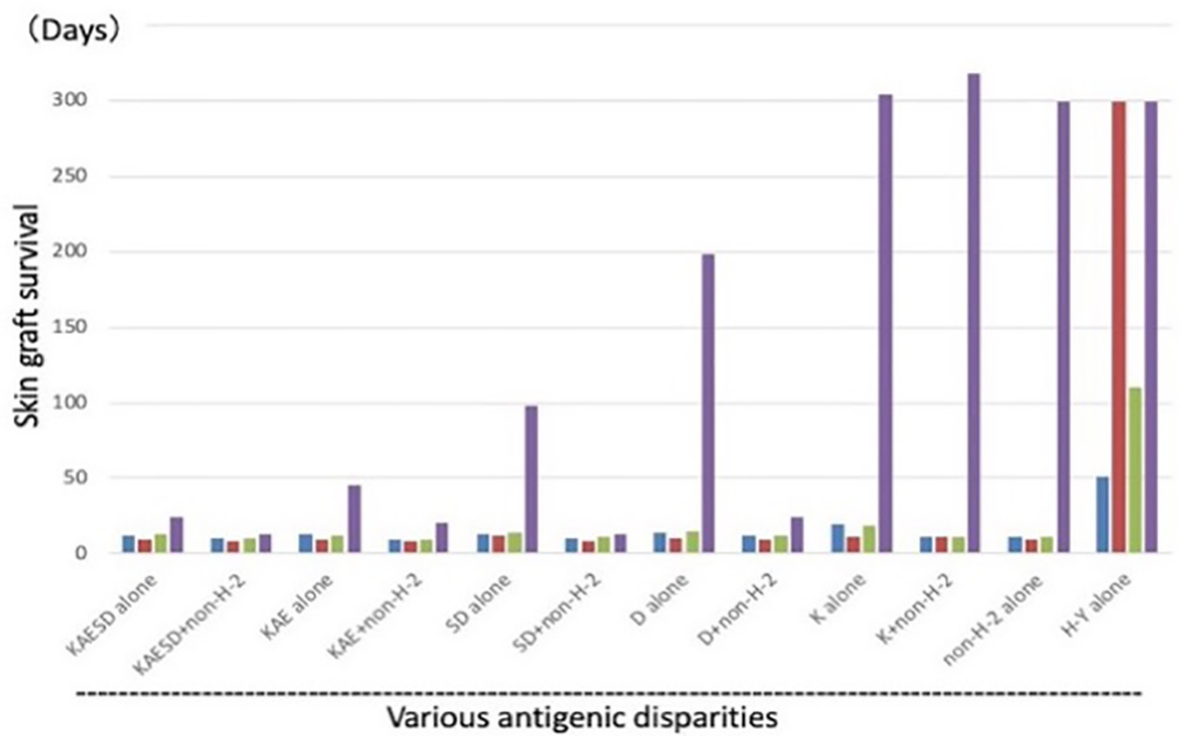
Tolerance induction across various allogeneic barriers; genetic analysis of sensitivity of tolerance induction using congenic strains. Using a variety of MHC-congenic strains, skin allograft tolerance induction was tried in various allogeneic combinations. Intravenous injection of 100 x 10^6^ allogeneic spleen cells on day -2 was followed by *i.p.* injection of 200 mg/kg Cy on day 0. Antigenic disparity between the recipient and donor was shown in the bottom. Since the murine MHC complex (H-2) consists of K, I-A, I-E, S, and D region genes (KAESD) with K, S, and D representing MHC Class I genes and I-A and I-E representing MHC Class II genes, the disparities were generated by variously combining the recipients and donors. The donor-recipient combinations were as follows: KAESD alone (B10.D2→B10), KAESD+non-H-2 (DBA→B10), KAE alone (B10.D2→B10.A), KAE+non-H-2 (DBA→B10.A), SD alone (B10.BR→B10.A), SD+non-H-2 (C3H→B10.A), D alone (B10.BR→B10.AKM), D+non-H-2 (C3H→B10.AKM), K alone (B6→B6.bm1), K+non-H-2 (C3H.SW→B6.bm1), non-H-2 alone (C3H.SW→B10), and H-Y alone (B10male→B10female). In each experimental group, untreated controls (blue bar), controls treated with spleen cells alone (red bar), and controls treated with Cy alone (green bar) were set up in addition to a tolerant (spleen cells + Cy; purple bar) group. Each bar represents mean skin graft survival time of 5-10 mice in each group. Skin grafting was performed on day 13. The abbreviations used for mice are as followed: AKR/J (AKR), BALB/cByJ (BALB), B6.C-H-2bm1 (B6.bm1), B10.AKM/SnJ (B10.AKM), B10.A/SgSnJ (B10.A), B10.BR/SgSnJ (B10.BR), B10.D2/SgSnJ (B10.D2), CBA/J (CBA), C3H/HeSnJ (C3H), C3H.SW/SnJ (C3H.SW), C57BL/6J (B6), C57BL/10SnJ (B10), and DBA/2J (DBA). Original data derived from reference (39).

In the Good lab, we first confirmed the necessity of both allogeneic antigens and stem cells for Cy-induced skin allograft tolerance in mice (38). Utilizing a wealth of congenic mouse strains and a murine skin allograft tolerance induction system that consists of intravenous injection of 100 x 10^6^ allogeneic SC followed by *i.p.* injection of 200 mg/kg Cy 2 days later, sensitivity to tolerance induction was examined across various H barriers (39) (**Figure 5**). Although each group of class I, class II or multi-minor H antigens was not by itself a prohibitively strong barrier, resistance to tolerance induction increased when the three types of barriers were combined in various ways (**Figure 5**). When the donor-recipient combinations were disparate at the entire spectrum of both H-2 plus non-H-2 antigens (KAESD+non-H-2(DBA→B10); Second group in **Figure 5**), profound tolerance to skin allografts was not induced by this method in any of the combinations examined (39).

c. Tolerance Induction Across Fully Allogeneic Barriers by the Two-Step Method

Based on these results, induction of tolerance across fully allogeneic barriers was attempted in C57BL/10SnJ (B10; H-2^b^) mice against C3H/HeSnJ (C3H; H-2^k^) strain by addressing the H barriers as two separate challenges (39). B10 mice were first given B10.BR/SgSnJ (B10.BR; H-2^k^) SC plus Cy to make them tolerant to the H-2^k^ component represented among C3H antigens, and then later were given C3H SC plus Cy to establish a tolerant state to the remainder of the disparate antigens of the C3H donors. After these two separate manipulations, C3H skin was accepted in the B10 mice, and normal hair growth was observed in the grafted C3H skin.

The important suggestion from the two-step method was that there were no specific loci generating the resistance to tolerance induction with cells-followed-by-Cy. The ease of tolerance induction appeared to be inversely related to the extent of antigen disparity between donor and recipient (39, 40). This might mean that the total number of reactive T cells in the recipients may be the key to succeed in the tolerance induction.

d. Long-Lasting Skin Allograft Tolerance Across Fully Allogeneic (Multimajor H-2 Plus Multiminor Histocompatibility) Antigen Barriers

**Figure 6:**
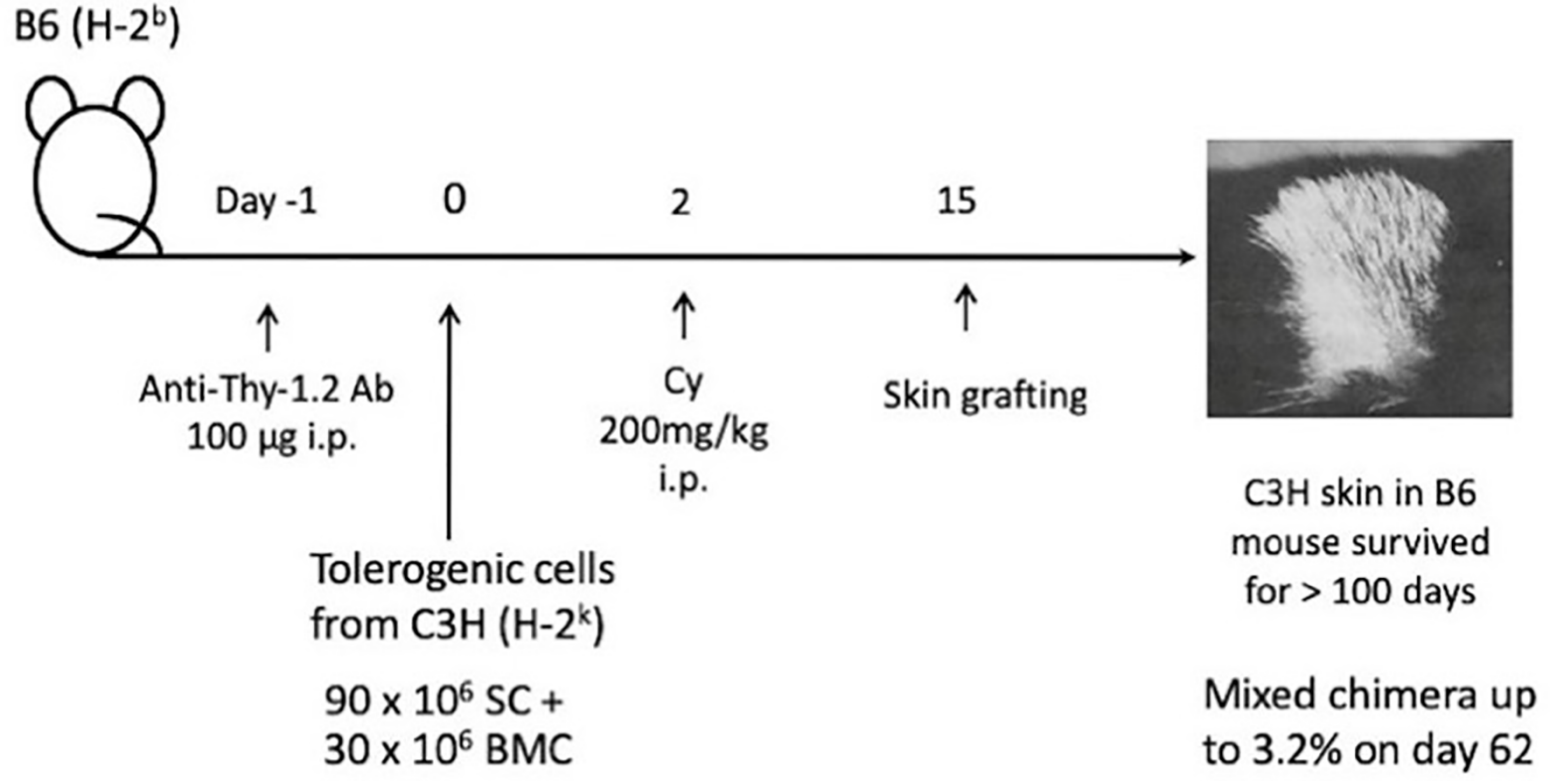
Long-lasting skin allograft tolerance in adult mice induced across fully allogeneic (multimajor H-2 plus multiminor histocompatibility) antigen barriers in various murine combinations. A method of Cy-induced skin allograft tolerance in mice that can regularly overcome fully allogeneic (major H-2 plus non-H-2) antigen barriers in mice was established. The components of the method are *i.p.* administration of 100μg of anti-Thy-1.2 monoclonal antibody (mAb) on day -1, *i.v.* injection of 90 x 10^6^ allogeneic SC mixed with 30 x 10^6^ allogeneic BMC from the same donor on day 0, and *i.p.* injection of 200 mg/kg Cy on day 2. In each of four fully allogeneic donor→recipient combinations, including C3H/HeJ (C3H; H-2^k^)→C57BL/6J(B6; H-2^b^), B6→C3H, BALB/cByJ (BALB; H-2^d^)→B6, and BALB→C3H, long-lasting survival of skin allografts is induced in most of the recipient mice. In the B6 mice made tolerant of C3H with mAb, C3H SC plus C3H BMC, and then Cy, a minimal degree of stable mixed chimerism up to 3.2% was established on day 62.

From the success of the two-step method in inducing skin tolerance across the strongest fully allogeneic barrier (39), we were strongly encouraged to go on to the next stage of devising a tolerance induction method. Consequently, a new method of Cy-induced skin allograft tolerance in mice that can regularly overcome fully allogeneic antigen barriers in mice was established (**Figure 6**) (41). The components of the method were *i.v.* or *i.p.* administration of 50-100μg of anti-Thy-1.2 mAb on day -1, *i.v.* injection of 90 x 10^6^ allogeneic SC mixed with 30 x 10^6^ allogeneic BM cells (BMC) from the same donor on day 0, and *i.p.* injection of 200 mg/kg Cy on day 2. In each of four fully allogeneic donor→recipient combinations (**Figure 6**), long-lasting survival of skin allografts was induced in most of the recipient mice (41). The optimal timing of Cy treatment to induce tolerance was found to be 1-3 days after the stimulating cell injection. In the B6 mice made tolerant of C3H with mAb, C3H SC plus C3H BMC, and then Cy, a minimal degree of stable mixed chimerism was established. From cell transfer experiments, the mechanism of tolerance could be largely attributed to reduction of effector T cells reactive against the tolerogen, whereas strong host anti-donor Ts activities that might prolong skin allograft survival directly were not detected in the tolerant mice (41).

These results suggest that permanent tolerance to fully allogeneic skin grafts may be induced because mAb given before the stimulating cell injection reduces the number of reactive T cells in the recipient mice. This mAb treatment may facilitate more complete clonal destruction. The injection of BMCs mixed with SCs appears to have facilitated maintenance of the tolerant state by establishing a state of stable mixed chimerism in the tolerant mice. In considering the present clinical setting, the preconditioning of the Johns Hopkins platform (**Figure 1**) may be somehow equivalent to this pretreatment with the mAbs.

e. Application of the Cy-Induced Tolerance System to Xenogeneic Combination of Rat→Mouse

**Figure 7:**
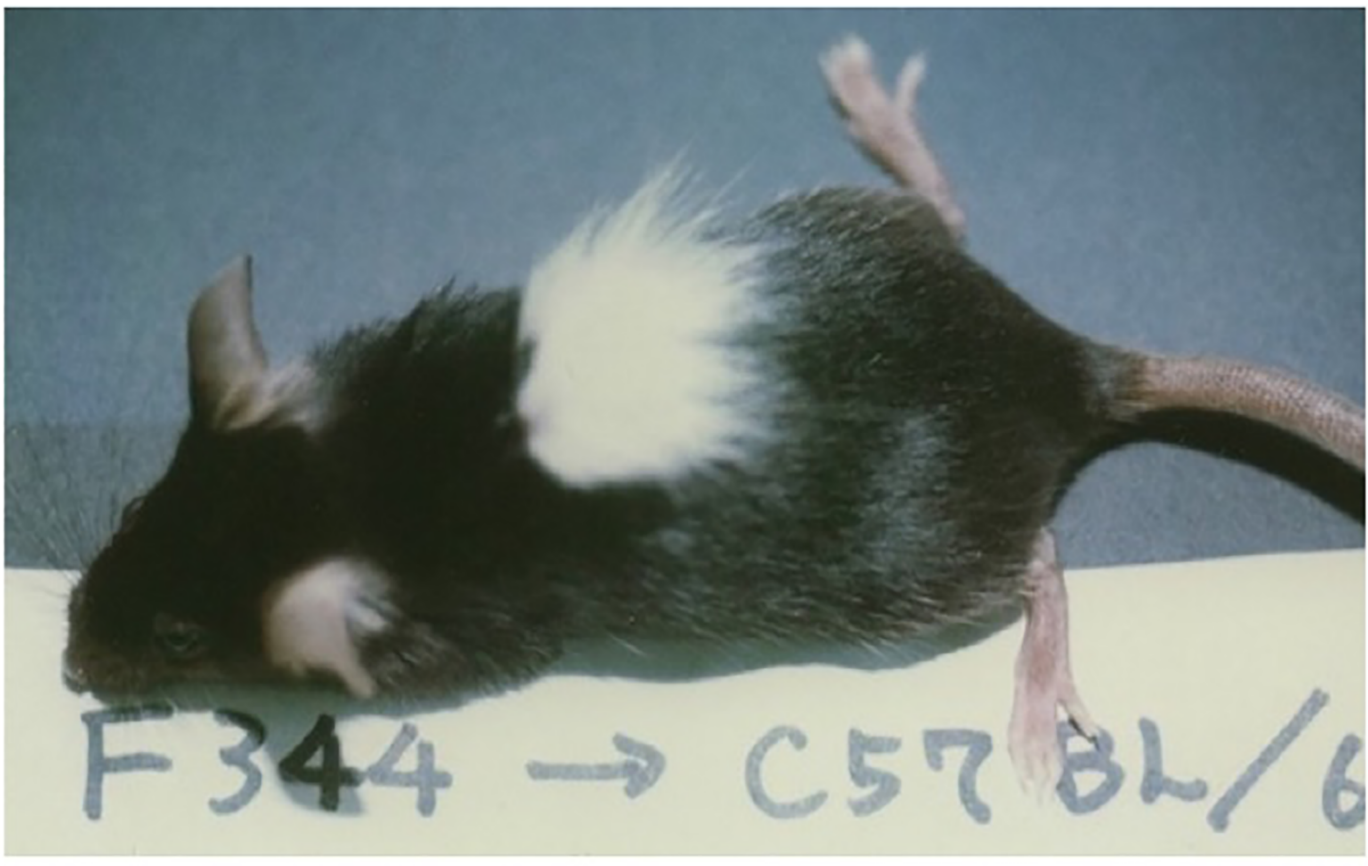
Skin xenograft tolerance induced in the B6 mice treated with anti-TCR-αβ mAb, anti-NK1.1 mAb, donor F344 cells, and Cy. The recipient B6 mouse was administered BMC (50×10^6^) and spleen cells (100×10^6^) from an F344 (RT^1^) rat on day 0, Cy (200mg/kg) on day 2, and anti-TCR-αβ mAb (H57-597, 400μg) and anti-NK1.1 mAb (PK136, 200μg) on days-1 and 3, and was grafted with F344 skin on day 14. The acceptance of F344 skin on day 50 is shown. The picture was kindly provided by Dr. Masayoshi Umesue.

Because of the success in inducing skin allograft tolerance across fully allogeneic (major H-2 plus non-H-2) antigen barriers (**Figure 6**) (41), we further investigated the possibility of inducing tolerance in a xenogeneic combination using Cy (42). Donor-specific prolongation of xenogeneic Fisher 344 (F344) rat (RT^1^) skin graft survival, associating with luxurious hair growth (**Figure 7**), for up to 60 days was induced in C57BL/6 (B6) mice by giving 50 x 10^6^ F344 bone marrow cells plus 100 x 10^6^ F344 spleen cells on day 0, 200mg/kg of Cy on day 2, and two mAbs against both murine TCR-αβ (H57-597, 400μg) and NK1.1 (PK136, 200μg) on days -1 and 3 (42). Here, the anti-TCR-αβ mAb was used in place of anti-Thy-1.2 mAb in the fully allogeneic system (**Figure 6**), and the anti-NK1.1 mAb was used because host NK cells were known to contribute to rejection of xenogeneic hematopoietic cells (43).

MLR, CTL activity, and antibody production against donor F344 were profoundly suppressed for 50 days in the tolerant recipient mice. After transplantation of donor F344 cells, mixed xenogeneic chimerism was observed in the spleen and peripheral blood of the tolerant B6 mice for 1 month, but was never observed in the thymus. Thus, neither intrathymic chimerism nor intrathymic clonal deletion was observed in the xenogeneic system. These results suggest that treatment with viable xenogeneic donor cells, Cy, and mAbs against T and NK cells can induce a temporary peripheral mixed chimerism and donor-specific prolongation of xenogeneic skin graft survival. The destruction with Cy of T and B cells that are xenoreactive and thus proliferating after antigen stimulation, followed by mechanism other than intrathymic clonal deletion, may be the mechanism of the hyporesponsiveness in the xeno system (42).

## Mechanisms of Cy-Induced Tolerance

The method we have used to prove the existence of the intrathymic clonal deletion was reported in a murine system by Kappler et al. (44, 45) and MacDonald et al. (46). So-called superantigens, such as Mls-1^a^ (the superantigen encoded by endogenous mammary tumor virus-7), can combine with MHC antigen class II molecules to form ligands that stimulate whole families of T cells *via* certain Vβ segments, such as Vβ6, of the TCR. Using the mAbs against these TCRs, T cells with such TCRs reactive to self Mls antigens are found in the immature population of thymocytes, but not in mature thymocytes or peripheral T cells pools. The T cells are thus deleted during their differentiation in the thymus. This system was first reported as a method to explain self-tolerance (44–46), but was soon used to explain allo-tolerance in neonatal tolerance system (47) followed by ours in Cy-induced tolerance system (48–55).

**Figure 8:**
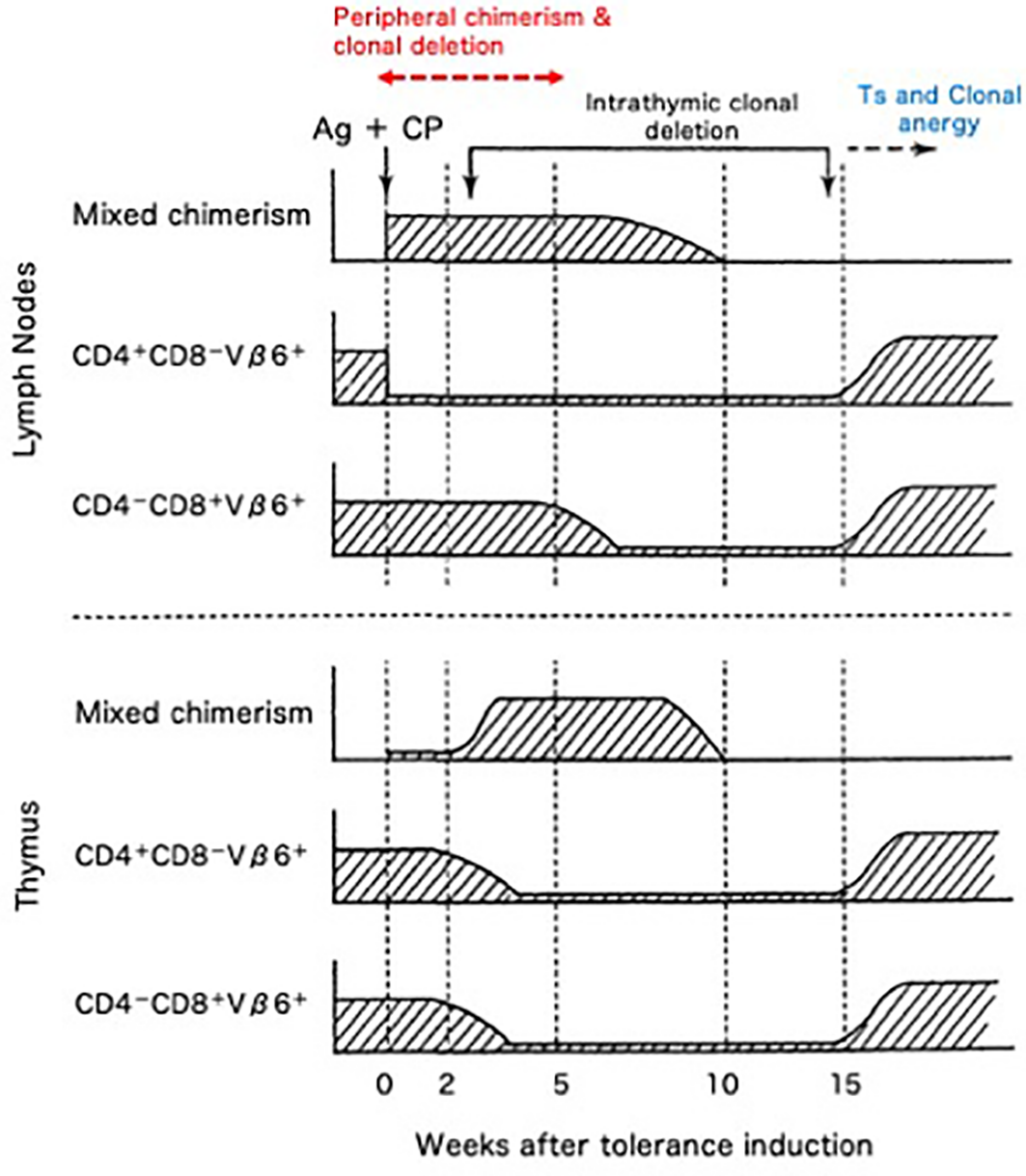
Cellular kinetics of Cy-induced skin allograft tolerance analyzed by using the monoclonal antibodies against superantigen-reactive T cells. The cellular kinetics that were obtained in the BALB/c (H-2^d^; Mls-1^b^) mice made tolerant of DBA/2 (H-2^d^; Mls-1^a^) with 100 x 10^6^ DBA/2 spleen cells followed by 200/kg Cy are summarized. The CD4^+^-Vβ6^+^ T cells that are responsible for the MLR against Mls-1^a^-encoded antigens and the effector T cells that are responsible for the rejection of DBA/2 skin were selectively destroyed (=clonal destruction) in the periphery (lymph nodes) of the tolerant BALB/c mice, leaving most of the non-proliferative CD8^+^-Vβ6^+^ T cells intact. The mixed chimeric state in the periphery was established right after the tolerance induction associating with peripheral clonal deletion (upper panel; =peripheral chimerism and peripheral clonal deletion). In the thymus, intrathymic mixed chimerism was gradually established because of the regeneration of the stem cells of donor origin contained in the tolerogenic spleen cells. At this stage, the clonal deletion of Vβ6^+^ T cells started to occur in the thymus (=intrathymic chimerism and intrathymic clonal deletion). The Vβ6^+^ cells, however, reappeared in the thymus and periphery, after the regression of the intrathymic mixed chimerism (lower panel). Even after the breakdown of the clonal deletion in the thymus, DBA/2-skin grafts were intact in the tolerant BALB/c mice. Both CD8^+^ suppressor T cells (=suppressor T cells) and clonal anergy (=clonal anergy) were considered to be responsible for maintaining the late stage of the tolerance.

The cellular kinetics of the transplantation tolerance in H-2 identical model systems of BALB/c (H-2^d^, Mls-1^b^) mice rendered Cy-induced tolerant to DBA/2 (H-2^d^, Mls-1^a^) skin allografts was investigated by assessing Vβ6^+^ T cells (48–55). From our results, five mechanisms that are essential to the Cy-induced skin allograft tolerance were elucidated (48–56) (**Figure 8**). The first mechanism was destruction of donor-antigen-stimulated-thus-clonally expanding mature T cells in the periphery by Cy treatment (48, 49, 51–54). Peripheral chimerism established by donor hematopoietic cell (usually SC) engraftment enables donor antigen-presenting cells to interact with, and induce the peripheral clonal deletion of, donor-reactive T cells (55) (=Second; peripherally induced clonal deletion). The third mechanism was intrathymic clonal deletion of donor-reactive T cells, such as Vβ6^+^ T cells, correlating strongly with intrathymic mixed chimerism (48–52). The clonal deletion, however, was not always essential for the maintenance of the skin allografts, because DBA/2 skin survived even after the intrathymic clonal deletion terminated and Vβ6^+^ T cells reappeared in the periphery of the recipient BALB/c mice (49). The fourth mechanism was generation of tolerogen-specific Ts cells, especially in the late stage of the tolerance (49, 56), although this activity was not detected in our earlier studies (3, 24, 25, 38, 39, 41, 57).

From all of our series of studies, the transfer experiments that were planned to examine the suppressor cell or serum activities were collected (**Table 1**). Here, in definition, the host Ts cell activity against donor skin graft antigens was always examined in our system, because the minimal degree of mixed chimerism established after the Cy-induced tolerance was always less than 5 percent. Seven experiments (3, 24, 25, 38, 39, 41, 57) out of the 9 studies done by various authors (3, 24, 25, 38, 39, 41, 49, 56, 57) could not detect any suppressor activities in the recipients given viable cells followed 2 days later by Cy at 4-57 days before the transfer experiments (Experiments 1-7). Both Y. Tomita et al. (56) (**Table 1**, Experiment 8-Groups 3 a, &c, and Groups 4 a, b, & c) and M. Eto et al. (49) (**Table 1**, Experiment 9-Group 1 b, and Groups 2 a, b, &c) could detect Ts activities in CD8^+^ T cells only when the transfer assays were done at the chronic phase of 84-100 days after the tolerance induction and only when the transferring recipient mice were pretreated with 300 cGy total body irradiation.

**Table 1:** Transfer experiments planned to examine the suppressor cell or serum activities by many researchers after inducing tolerance in mice with allogeneic cells followed by Cy.

Experiment	Primary author (reference)	Group		Abrogation or generation of tolerance by transfering with				Disparities	Allograft	Results	Comments
				Transferred donor cells or serum	Timing of transfer	Pretreatment of recipients	Recipients				
1	Shin T (24)	a		Naive AKR SC	4 days after Cy	None	AKR mice tolerant to B6	Major H-2 + Minor	EL-4 tumor allograft	Rejected	Breakdown of tolerance with naive SC
		b		SC from AKR mice tolerant to B6	4 days after Cy	None	Naive AKR mice	Major H-2 + Minor	EL-4 tumor allograft	Not prolonged	Failure of transfering tolerance using tolerant SC into naive recipients
		c		Serum from AKR mice tolerant to B6	4 days after Cy	None	Naive AKR mice	Major H-2 + Minor	EL-4 tumor allograft	Not prolonged	Failure of transfering tolerance using tolerant serum into naive recipients
2	Mayumi H(3)	a		SC from naive C3H mice	7 days after Cy	None	C3H mice tolerant to AKR	Minor alone	AKR skin graft	Normaly rejected	Breakdown of tolerance with naive SC
		b		T cell-depleted(=anti-θ+C) SC from naive C3H mice	7 days after Cy	None	C3H mice tolerant to AKR	Minor alone	AKR skin graft	No rejection	Sustained tolerance
		c		SC from C3H mice tolerant to AKR	7 days after Cy	None	Naive C3H mice	Minor alone	AKR skin graft	Not prolonged	Failure of transfering tolerance using tolerant SC into naive recipients
3	Mayumi H(57)	a		SC from C3H mice made tolerant to AKR with AKR SC+Cy	57 days after Cy	None	Naive C3H mice	Minor alone	AKR skin graft	Not prolonged	Failure of transfering tolerance using tolerant SC into naive recipients
		b		SC from C3H mice made tolerant to AKR with Atx+AKR SC+Cy	57 days after Cy	None	Naive C3H mice	Minor alone	AKR skin graft	Not prolonged	Failure of transfering tolerance using tolerant SC into naive recipients
		c		LNC from C3H mice made tolerant to AKR with Splx+AKR SC+Cy	57 days after Cy	None	Naive C3H mice	Minor alone	AKR skin graft	Not prolonged	Failure of transfering tolerance using tolerant LNC into naive recipients
4	Tokuda N(25)	a		SC from C3H made tolerant to B6 by in vitro presensitization with MMC treated B6 cells for 48hr followed by 9hr treatment with 5-FU	Soon after 5-FU treatment	None	Naive C3H SC	Major H-2 + Minor	% Cytotoxicity to B6 Con A blasts	Regained in *in vitro* cytotoxicity	Breakdown of tolerance with naive SC
		b		Naive C3H SC	Soon after 5-FU treatment	None	SC from C3H made tolerant to B6 by *in vitro* presensitization with MMC treated B6 cells for 48hr followed by 9hr treatment with 5-FU	Major H-2 + Minor	% Cytotoxicity to B6 Con A blasts	No decrease in *in vitro* cytotoxicity	Failure of transfering *in vitro* tolerance using tolerant SC into naive SC
5	Mayumi H(38)	a		SC from naive C3H mice	14 days after Cy	None	C3H mice made tolerant to AKR with AKR iSC+BMC+Cy	Minor alone	AKR skin graft	Normaly rejected	Breakdown of tolerance with naive SC
		b		SC from C3H mice made tolerant to AKR with AKR iSC+BMC+Cy	14 days after Cy	None	Naive C3H mice	Minor alone	AKR skin graft	Not prolonged	Failure of transfering tolerance using tolerant SC into naive recipients
		c		Serum from C3H mice made tolerant to AKR with AKR iSC+BMC+Cy	14 days after Cy	None	Naive C3H mice	Minor alone	AKR skin graft	Not prolonged	Failure of transfering tolerance using tolerant serum into naive recipients
6	Mayumi H(39)	a		SC from naive B10 mice	14 days after second Cy	None	B10 mice made tolerant to C3H with two-step method (B10.BR SC+Cy→C3H SC+Cy)	Major H-2 + Minor	C3H skin graft	Normaly rejected	Breakdown of tolerance with naive SC
		b		SC from B10 mice made tolerant to C3H with two-step method (B10.BR SC+Cy→C3H SC+Cy)	14 days after second Cy	None	Naive B10 mice	Major H-2 + Minor	C3H skin graft	Not prolonged	Failure of transfering tolerance using tolerant SC into naive recipients
		c		Serum from B10 mice made tolerant to C3H with two-step method (B10.BR SC+Cy→C3H SC+Cy)	14 days after second Cy	None	Naive B10 mice	Major H-2 + Minor	C3H skin graft	Not prolonged	Failure of transfering tolerance using tolerant serum into naive recipients
7	Mayumi H (41)	a		SC from naive B6 mice	15 days after Cy	None	B6 mice made tolerant to C3H with anti-Thy1.2 Ab/C3H SC+BMC/Cy	Major H-2 + Minor	C3H skin graft	Normaly rejected	Breakdown of tolerance with naive SC
		b		SC from B6 mice made tolerant to C3H with anti-Thy1.2 Ab & C3H SC+BMC & Cy	15 days after Cy	None	Naive B6 mice	Major H-2 + Minor	C3H skin graft	Not prolonged	Failure of transfering tolerance using tolerant SC into naive recipients
		c		Serum & SC from B6 mice made tolerant to C3H with anti-Thy1.2 Ab & C3H SC+BMC & Cy	15 days after Cy	None	Naive B6 mice	Major H-2 + Minor	C3H skin graft	Not prolonged	Failure of transfering tolerance using tolerant serum+SC into naive recipients
8	Tomita Y(56)	1	a	SC from naive AKR mice	14 days after Cy	None	AKR mice made tolerant to C3H with C3H SC+BMC/Cy	Minor alone	C3H skin graft	Normaly rejected	Breakdown of tolerance with naive SC
			b	SC from naive AKR mice	14 days after Cy	None	AKR mice made tolerant to C3H with C3H+B6(SC+BMC)/Cy	Minor alone	C3H skin graft	Normaly rejected	Breakdown of tolerance with naive SC
			c	SC from naive AKR mice	14 days after Cy	None	AKR mice made tolerant to C3H with B6C3F1(SC+BMC)/Cy	Minor alone	C3H skin graft	Normaly rejected	Breakdown of tolerance with naive SC
		2	a	SC from AKR mice made tolerant to C3H with C3H SC+BMC/Cy	14 days after Cy	None	Naive AKR mice	Minor alone	C3H skin graft	Not prolonged	Failure of transfering tolerance using tolerant SC into naive recipients
			b	Serum from AKR mice made tolerant to C3H with C3H SC+BMC/Cy	14 days after Cy	None	Naive AKR mice	Minor alone	C3H skin graft	Not prolonged	Failure of transfering tolerance using tolerant serum into naive recipients
			c	SC from AKR mice made tolerant to C3H with C3H+B6(SC+BMC)/Cy	14 days after Cy	None	Naive AKR mice	Minor alone	C3H skin graft	Not prolonged	Failure of transfering tolerance using tolerant SC into naive recipients
			d	Serum from AKR mice made tolerant to C3H with C3H+B6(SC+BMC)/Cy	14 days after Cy	None	Naive AKR mice	Minor alone	C3H skin graft	Not prolonged	Failure of transfering tolerance using tolerant serum into naive recipients
			e	SC from AKR mice made tolerant to C3H with B6C3F1(SC+BMC)/Cy	14 days after Cy	None	Naive AKR mice	Minor alone	C3H skin graft	Not prolonged	Failure of transfering tolerance using tolerant SC into naive recipients
			f	Serum from AKR mice made tolerant to C3H with B6C3F1(SC+BMC)/Cy	14 days after Cy	None	Naive AKR mice	Minor alone	C3H skin graft	Not prolonged	Failure of transfering tolerance using tolerant serum into naive recipients
		3	a	SC from AKR mice made tolerant to C3H with C3H SC+BMC/Cy	84 days after Cy	None	Naive AKR mice	Minor alone	C3H skin graft	Slightly prolonged	Partial success in transfering tolerance using tolerant SC into naive recipients
			b	Serum from AKR mice made tolerant to C3H with C3H SC+BMC/Cy	84 days after Cy	None	Naive AKR mice	Minor alone	C3H skin graft	Not prolonged	Failure of transfering tolerance using tolerant serum into naive recipients
			c	SC from AKR mice made tolerant to C3H with C3H+B6(SC+BMC)/Cy	84 days after Cy	None	Naive AKR mice	Minor alone	C3H skin graft	Slightly prolonged	Partial success in transfering tolerance using tolerant SC into naive recipients
			d	Serum from AKR mice made tolerant to C3H with C3H+B6(SC+BMC)/Cy	84 days after Cy	None	Naive AKR mice	Minor alone	C3H skin graft	Not prolonged	Failure of transfering tolerance using tolerant serum into naive recipients
			e	SC from AKR mice made tolerant to C3H with B6C3F1(SC+BMC)/Cy	84 days after Cy	None	Naive AKR mice	Minor alone	C3H skin graft	Not prolonged	Failure of transfering tolerance using tolerant SC into naive recipients
			f	Serum from AKR mice made tolerant to C3H with B6C3F1(SC+BMC)/Cy	84 days after Cy	None	Naive AKR mice	Minor alone	C3H skin graft	Not prolonged	Failure of transfering tolerance using tolerant serum into naive recipients
		4	a	C' treated SC from AKR mice made tolerant to C3H with C3H SC+BMC/Cy	84 days after Cy	300 rad	Irradiated AKR mice	Minor alone	C3H skin graft	Comparatively prolonged	Success in transfering tolerance using tolerant SC into naive recipients
			b	Anti-Thy1.1 Ab+C' treated SC from AKR mice made tolerant to C3H with C3H SC+BMC/Cy	84 days after Cy	300 rad	Irradiated AKR mice	Minor alone	C3H skin graft	Slightly prolonged	T cells are resposible for transfering tolerance using tolerant SC into naive recipients
			c	Anti-CD8 Ab+C' treated SC from AKR mice made tolerant to C3H with C3H SC+BMC/Cy	84 days after Cy	300 rad	Irradiated AKR mice	Minor alone	C3H skin graft	Slightly prolonged	CD8+ T cells are resposible for transfering tolerance using tolerant SC into naive recipients
9	Eto M (49)	1	a	SC from DBA mice made tolerant to BALB with BALB SC/Cy	14 days after Cy	300 rad	Irradiated BALB mice	Minor alone	DBA skin graft	Not prolonged	Failure of transfering tolerance using tolerant SC into naive recipients
			b	SC from DBA mice made tolerant to BALB with BALB SC/Cy	100 days after Cy	300 rad	Irradiated BALB mice	Minor alone	DBA skin graft	Comparatively prolonged	Success in transfering tolerance using tolerant SC into naive recipients
		2	a	C' treated SC from DBA mice made tolerant to BALB with BALB SC/Cy	100 days after Cy	300 rad	Irradiated BALB mice	Minor alone	DBA skin graft	Comparatively prolonged	Success in transfering tolerance using tolerant SC into naive recipients
			b	Anti-Thy1.1 Ab+C' treated SC from DBA mice made tolerant to BALB with BALB SC/Cy	100 days after Cy	300 rad	Irradiated BALB mice	Minor alone	DBA skin graft	Slightly prolonged	T cells are resposible for transfering tolerance using tolerant SC into naive recipients
			c	Anti-CD8 Ab+C' treated SC from DBA mice made tolerant to BALB with BALB SC/Cy	100 days after Cy	300 rad	Irradiated BALB mice	Minor alone	DBA skin graft	Slightly prolonged	CD8+ T cells are resposible for transfering tolerance using tolerant SC into naive recipients

From all of our series of studies, the transfer experiments that were planned to examine the suppressor cell or serum activities were collected. Here, in definition, the host Ts cell activity against donor skin graft antigens was always examined in our system, because the minimal degree of mixed chimerism established after the Cy-induced tolerance was always less than 5 percent. Seven experiments (3, 24, 25, 38, 39, 41, 57) out of the 9 studies done by various authors (3, 24, 25, 38, 39, 41, 49, 56, 57) could not detect any suppressor activities in the recipients given viable cells followed 2 days later by Cy at 4-57 days before the transfer experiments (Experiments 1-7). Both Y. Tomita et al. (56); **Table 1**, Experiment 8-Groups 3 a, &c, and Groups 4 a, b, &c) and M. Eto et al. (49); **Table 1**, Experiment 9-Group 1 b, and Groups 2 a, b, &c) could detect suppressor cell activities in CD8^+^ T cells only when the transfer assays were done at the chronic phase of 84-100 days after the tolerance induction and only when the transferring recipient mice were pretreated with 300 rad irradiations.

AKR, AKR/J (H-2^k^); B6, C56BL/6 (H-2^b^); C3H, C3H/HeN (H-2^k^); B10, C57BL/10SnJ (H-2^b^); B10.BR, B10.BR/SgSnJ (H-2^k^); B6C3F1, C57BL/6×C3H/HeN F1 (H-2^b+k^); DBA, DBA/2J (H-2^d^); BALB, BALB/cByJ (H-2^d^).

SC, spleen cells; Cy, cyclophosphamide; Atx, adult thymectomy; LNC, lymph node cells; MMC, mitomycin C; 5-FU, fluorouracil; iSC, irradiated spleen cells; BMC, bone marrow cells.

The clonal anergy (the immune system is unable to mount a normal immune response against a specific antigen) reported by Rammensee HG et al. (58) was not considered as a significant mechanism in our system at first, but Y. Tomita et al. (59), by using a class I (D region)+Class II (IE region) disparate donor→recipient combination of B10. A (5R) (K^b^, IA^b^, IE^b^, D^d^; Thy-1.2)→B10.Thy-1.1 (K^b^, IA^b^, IE^-^, D^b^; Thy-1.1) mice and the mAb against Vβ11 and Vβ5 TCR, demonstrated that clonal anergy is one of the important mechanisms of the Cy-induced tolerance (=fifth; clonal anergy) after the termination of intrathymic clonal deletion (59).

## Discussion

From our basic studies (3, 28), BMT/PTCy-induced tolerance would seem most suited to HLA-matched combinations. However, BMT/PTCy was not applied initially to HLA-matched transplants because conventional GVHD prophylaxis such as tacrolimus plus methotrexate was enough to achieve acceptably low incidences of GVHD and graft rejection. Instead, the first clinical trial in BMT examined haplo-identical combinations. Based on the promising results of PTCy in haploBMT (haploBMT/PTCy), many groups now use the same strategy in the setting of HLA-matched donors because of its safety, simplicity, and low cost (60–63).

Although chronic GVHD after BMT is a definite problem, it is beneficial for suppressing the recurrence of the malignant hematopoietic diseases. This phenomenon is called the GVL effect (64, 65). Allogeneic lymphocytes produce a strong GVL effect, but the beneficial effect is offset by GVHD when it is too strong. Depletion of T cells, in order to abrogate GVHD and GVL effects, and delayed transfusion of donor lymphocytes into chimeras after T cell-depleted stem cell transplantation produces a GVL effect without necessarily producing GVHD (65). PTCy, however, is probably providing a clinically significant GVL effect without performing such complex procedures. Since the H mismatches in the HLA-haploidentical PTCy appear to be 1-3 HLA plus minor H antigens, a certain amount of the split tolerance mechanism works (**Figure 4**). A part of mature T cells in the recipient appeared to be less proliferative against the antigen stimulation, mature quickly before the Cy-treatment, and thus remain in an anamnestic state after the Cy-treatment. The more an antigenic disparity between donor and host exists, the larger number of sensitized cells seems to remain after tolerance induction by the cells-followed-by-drug system (8–10, 24). The existence of such anamnestic cells in the tolerant recipient (or cultured cells) is manifested as a form of split tolerance (8–10, 24, 25, 34–36). In the haploBMT patients treated with PTCy, therefore, the small amount of GVL effect occurs naturally, depending on the H antigen disparity.

As discussed above, the state of split tolerance typically generated after the induction of tolerance between fully allogeneic combinations (**Figure 4**) might result in GVL activities. This possibility may explain unexpected phenomena in clinical setting. For example, from the results of the single-agent GVHD prophylaxis with PTCy after myeloablative, HLA-matched BMT for malignant hematopoietic diseases (62), the cumulative incidences of both acute GVHD and chronic GVHD were higher in the HLA-matched-unrelated allografting than in the HLA-matched-related allografting. The non-relapse mortality, however, was equal between these two groups and the relapse appeared to be less in the HLA-matched-unrelated allografting (62). This fact may also suggest that the larger H antigenic disparity, including HLA-DPB1 mismatching which occurs frequently in unrelated donor transplants (66), may not necessarily result in a worse consequence after BMT with PTCy. This may be also because of the nature of the split tolerance and its GVL effect. Generally speaking, however, for patients with hematological malignancies who underwent haploidentical transplantation based on Cy induced tolerance, the cumulative incidences of relapse at 1 year were more than 50%. In another haplo-BMT/PTCy protocol (67, 68), the GVL effect was superior to that of HLA-matched sibling donor transplantation (67, 68). The strength of rejection, moreover, is as strong as rejecting a skin allograft or the small doses of tumor allografts but not the large doses of tumor allografts from our old studies (27, 34, 36). That is; the residual alloreactivity (=GVL effects) after PTCy must be marginal and GVL activity may be proportional to the antigenic disparity between donor and host. Therefore, all of the residual malignancy after the preconditioning is not always and completely destroyed by this power. Only when the cumulative GVL effect (evaluated by the absence of relapse, for example) is compared in the HLA-matched-unrelated allografting and in the HLA-matched-related allografting (=with subtle difference), the marginal effect may be revealed.

In order to explain the phenomenon of the split tolerance that is generated after allogeneic SC injection followed 1-3 days later by bolus Cy, I have proposed the idea of the less proliferative quick maturation of the antigen-stimulated mature T cells during the 1-3 days interval (36) (**Figure 4**): that is, the resistance to the clonal destruction. Although this phenomenon may be partially explained as a cross reactivity of the allo H antigens after viral infection (69) as was discussed by E. J. Fuchs (2), the remained anamnestic allo-antigen reactivity is antigen specific and could not be augmented by the further immunization with the tolerogenic allo-antigens (34). Until recently, the understanding of TCR recognition of peptide (70) seems to be complex and there are at least three patterns of T-cell recognition: a) molecular mimicry (71), b) induced fit (72) and c) disparate docking (73). Some of such allo-recognition models may explain the less-proliferative quick maturation and generation of the split tolerance, but should be further studied in detail. This mechanism seems to be much more important for GVL than previously considered.

As for the clinical interval between the hematopoietic cell injection and following Cy administration, 50mg/kg of Cy was given on day 3 alone in the nonmyeloablative BMT from partially HLA-mismatched related donors using PTCy in the initial report from the Johns Hopkins group (74). This timing appeared to be decided from the basic murine (B10.BR (H-2^k^)→B10 (H-2^b^))study performed by L. Luznik et al. (4) by obtaining a hint from our timing study in H-2-identical murine combinations (3). Another Cy injection of 50mg/kg on day 4 was soon added to reduce both engraftment failure and severe acute GVHD (75). To my knowledge, however, this additional Cy administration timing on day 4 among the widely spread PTCy methods is empirical to the last. My colleague Zhang had clearly shown that fractionated Cy is effective in ameliorating the compromised state induced by a single dose of 200 mg/kg Cy, but is divided into three or fewer fractions by giving Cy at 100 and 66 mg/kg daily from day 1 through days 2 and 3 (76). The best timing of single Cy administration has been 1-3 days after the antigenic cell injection no matter how the antigenic disparity changes. This was also the case *in vitro* using 5-fluorouracil in place of Cy (25, 35). I was astonished to see that the recent study not only in clinic but also in a murine system (6) employed the timing of Cy treatment on days 3 and 4 to induce tolerant state. In the clinical haploBMT/PTCy, therefore, the best timing of the drug-treatment should be reconfirmed in clinical trials, or at least *in vitro* by using our 5-fluorouracil-induced *in vitro* tolerance system (25, 35) in humans.

A recent report by L.P. Wachsmuth et al. described that PTCy did not eliminate alloreactive T cells and the thymus was not necessary for the efficacy of PTCy in the MHC-haploidentical model of B6C3F1→10.5 Gy-irradiated B6D2F1, whereas the rapid recovery of Tregs expressing aldehyde dehydrogenase played an important role in suppressive mechanisms of GVHD by PTCy (12). From the entire review of our series of studies, the transfer experiments that were planned to examine the host suppressor cell or serum activities were collected and listed in **Table 1**. Seven experiments (3, 24, 25, 38, 39, 41, 57) out of the 9 studies done by various authors (3, 24, 25, 38, 39, 41, 49, 56, 57) could not detect any host suppressor activities against donor antigens in the recipients given viable cells followed 2 days later by Cy at 4-57 days before the transfer experiments (**Table 1**). Both Y. Tomita et al. (56) and M. Eto et al. (49) could detect suppressor cell activities in CD8^+^ T cells only when the transfer assays were done at the chronic phase of 84-100 days after the tolerance induction and only when the transferring recipient mice were pretreated with 300 rad irradiations. Generation of Ts cell activities may be related to the irradiated recipient mice as was seen in Wachsmuth’s experiments (13). Besides, the elegant five mechanisms of Cy-induced tolerance including Ts activities work only when the donor-recipient combination is H-2 identical. In the murine combination of B6C3F1→B6D2F1 model and in the human haploBMT/PTCy situation, the clonal destruction may be imperfect because of the partial MHC plus minor H antigen disparities resulting in the split tolerance (**Figure 4**). Such a situation may permit the sudden generation of Ts cells after Cy-treatment.

The difficulty in inducing Ts cell activities in our systems but neither in Kanakry’s system or Johns Hopkins haploBMT/PTCy system may be alternatively explained as volume of antigens to-be-rejected. In our Cy-induced tolerance systems in H-2-matched murine combinations, the level of mixed chimerism is usually less than 5%. The measured Ts cells were almost all host origin. The donor cells to be rejected by the host lymphocytes in this case is at most 5% of all leukocytes. In contrast, the Ts cell activities measured after BMT/PTCy must be almost all donor origin because of the successful establishment of complete chimerism. For the donor-originated lymphocytes including the measured Ts cells, the antigen to-be-rejected is the entire host body tissues. In the latter case, a stronger Ts cell activity may be generated sooner and stronger.

As was shown in the xeno-tolerance system (**Figure 7**), the failure in establishing stable intrathymic chimerism appears to be the cause of the moderate success in the rat→mouse system (42). This small degree of mixed chimerism may be considered to be the cause of the hardness in generating host-originated suppressor T cell activities in our systems (**Table 1**). When the stage is changed from the “Cy-induced tolerance alone” to the “haploBMT/PTCy” by using the Johns Hopkins Platform (preconditioning+BMT/PTCy+post-immunosuppression; **Figure 1**), the minimum degree of mixed chimerism is changed to the complete chimerism. The volume of antigens to-be-rejected is changed from less than 5% of donor-originated lymphocytes to the recipient entire body. In this situation, probably a strong donor-originated Ts cell activity is generated, and thus the remaining less-proliferative memory T cells after BMT/PTCy in the partially HLA-mismatched donor-recipient combinations could be the mild effectors of GVL effect but could not be the strong effectors of GVHD. Another study showed that anti-donor Ts cells are generated when a low dose of BMC is given under the cover of non-depleting anti-CD4 (and depleting anti-CD8) mAbs (77). Whereas host anti-donor CD4^+^ T cells may have developed into Ts cells in that system, this process may be prevented by clonal destruction in the cells-followed-by-Cy system. As was shown in our previous study (30), the Cy-induced tolerance per se is established so as to suppress GVHD soon after the SC plus Cy-treatment by the mechanism of clonal destruction (30).

As the perspective of Cy-induced tolerance in clinic, it may be possible to use the method devised for the fully allogeneic systems (that is; anti-T cell mAb+BMCs+Cy). But so far, there are no patients who obtained direct benefits from solid organ transplantation based on Cy-induced tolerance because no clinical trials have used this system. In the tolerance induction by the BMT/PTCy, the residual small reactivity in the partially HLA-mismatched donor-recipient combinations may be beneficial in generating GVL effects and strong Ts activities. Therefore, the system may be used in wide-range of donor-recipient combinations. The Cy-induced tolerance itself, however, may be usable in limited donor-recipient combinations such as in the HLA-matched-related or the HLA-matched-unrelated allografting.

## Author Contributions 

The author confirms being the sole contributor of this work and has approved it for publication.

## Conflict of Interest

The author declares that the research was conducted in the absence of any commercial or financial relationships that could be construed as a potential conflict of interest.

## Publisher’s Note

All claims expressed in this article are solely those of the authors and do not necessarily represent those of their affiliated organizations, or those of the publisher, the editors and the reviewers. Any product that may be evaluated in this article, or claim that may be made by its manufacturer, is not guaranteed or endorsed by the publisher.
